# Antibiotic-resistant *Escherichia coli* from treated municipal wastewaters and Black-headed Gull nestlings on the recipient river

**DOI:** 10.1016/j.onehlt.2024.100901

**Published:** 2024-09-22

**Authors:** Martina Masarikova, Iva Sukkar, Ivana Jamborova, Matej Medvecky, Ivo Papousek, Ivan Literak, Alois Cizek, Monika Dolejska

**Affiliations:** a Department of Infectious Diseases and Microbiology, Faculty of Veterinary Medicine, University of Veterinary Sciences Brno, Brno, Czech Republic; b Central European Institute of Technology, University of Veterinary Sciences Brno, Brno, Czech Republic; c Biomedical Center, Faculty of Medicine, Charles University, Pilsen, Czech Republic; d Department of Biology and Wildlife Diseases, Faculty of Veterinary Hygiene and Ecology, University of Veterinary Sciences Brno, Brno, Czech Republic; e Division of Clinical Microbiology and Immunology, Department of Laboratory Medicine, The University Hospital Brno, Czech Republic

**Keywords:** Enterobacterales, Environment, Wild birds, Whole-genome sequencing

## Abstract

Wastewaters belong among the most important sources of environmental pollution, including antibiotic-resistant bacteria. The aim of the study was to evaluate treated wastewaters as a possible transmission pathway for bacterial colonisation of gulls occupying the receiving river. A collection of antibiotic-resistant *Escherichia coli* originating both from treated municipal wastewaters discharged to the river Svratka (Czech Republic) and nestlings of Black-headed Gull (*Chroicocephalus ridibundus*) living 35 km downstream of the outlet was obtained using selective cultivation. Isolates were further characterised by various phenotyping and genotyping methods.

From a total of 670 *E. coli* isolates (450 from effluents, 220 from gulls), 86 isolates (41 from effluents, 45 from gulls) showed identical antibiotic resistance phenotype and genotype and were further analysed for clonal relatedness using pulsed-field gel electrophoresis (PFGE). Despite the overall high diversity of the isolates, 21 isolates from both sources showed similar PFGE profiles. Isolates belonging to epidemiologically important sequence types (ST131, 15 isolates; ST23, three isolates) were subjected to whole-genome sequencing. Subsequent phylogenetic analysis did not reveal any close clonal relationship between the isolates from the effluents and gulls' nestlings with the closest strains showing 90 SNPs difference.

Although our study did not provide direct evidence of transmission of antibiotic-resistant *E. coli* to wild gulls via treated wastewaters, we observed gull chicks as carriers of diverse multi-resistant *E. coli*, including high-risk clones, posing risk of further bacterial contamination of the surrounding environment.

## Highlights

• Molecular characterization of resistant *E. coli* from wastewater effluent and gulls.
• Treated effluents contain multi-resistant *E. coli* despite the treatment process.
• The high-risk clone *E. coli* ST131 was detected among gull's nestlings.
• Limited relatedness among *E. coli* strains observed between gulls and effluents.

## 1 Introduction

Wastewater treatment plant (WWTP) effluents can serve as one of the most important sources of antibiotic-resistant bacteria for ecosystems including surface waters [1,2]. Rivers receiving the insufficiently treated effluents then represent effective vehicles for spreading of resistant microorganisms over long distances. They are also a medium where bacteria of human and animal origin are brought into contact with naturally occurring bacteria of the aquatic ecosystem, thus enabling the exchange of genetic material and creation of new environmental reservoirs of antibiotic resistance genes [[3], [4], [5]].

Gulls live in close contact with aquatic environments and therefore can become colonised by antibiotic-resistant enterobacteria via treated wastewater effluents [6,7]. *E. coli* resistant to cephalosporins and carbapenems has been previously documented in gulls from a variety of places, e.g. Czech Republic, France, Portugal, Sweden, United States of America, Canada, Australia, Russia or Alaska [8]. Gulls are considered to play a role in the dissemination of intestinal pathogens not only because of their migratory behaviour but also because of their large populations and worldwide distribution [9]. In our previous work, epidemiological relatedness was observed between antibiotic-resistant *Salmonella* spp. isolates originating from wastewater effluents and nestlings of gulls from a breeding colony at recipient river [10]. Here, we broaden the preceding findings with a collection of *E. coli* resistant to beta-lactams and quinolones obtained from the same samples of wastewater effluents and nestlings of gulls. To identify their epidemiological association, and hence the possibility of gulls' colonisation with resistant *E. coli* of wastewater origin, the clonal relationship between isolates originating from these two sources was evaluated using antibiotic susceptibility testing, genotyping and whole-genome sequencing (WGS).

## 2 Material and methods

### 2.1 Samples collection

Effluents of treated wastewaters were sampled at the discharge from the municipal WWTP (Brno, Czech Republic; 49°8′N 16°38′E) which is flowing into river Svratka. The WWTP processes wastewaters from the city of Brno (approximately 400.000 inhabitants) and surrounding area using a two-stage treatment [11]. A total of 37 effluent samples were collected weekly between March and December 2012 using Moore cotton swabs. Swabs were placed at the effluent for one week and then transported in sterile containers to the laboratory for cultivation.

Chicks of Black-headed Gull (*Chroicocephalus ridibundus*) were one-time sampled in the breeding colony at Nove Mlyny dam (48°53′N 16°36′E) situated 35 km downriver of the WWTP in May 2012 simultaneously with bird ringing. It is a period when most of the nestlings in the colony have hatched, but they are not developed enough to fly. A total of 284 cloacal swabs were collected (one sample representing one individual bird) and processed for cultivation.

### 2.2 Isolation and identification of antibiotic-resistant *E. coli*

Samples were cultivated in buffered peptone water overnight [10] and subcultured on MacConkey agar (MCA) containing cefotaxime (MCA_cef_, 2 mg/L) and on MCA with ciprofloxacin (MCA_cip_, 0.05 mg/L). In the case of gull samples (G), one lactose-positive colony of presumptive *E. coli* isolates was collected. For treated wastewater effluent (WE) samples, where a more diverse spectrum of *E. coli* of different origins can be expected, a maximum of 10 lactose-positive colonies were taken. Obtained colonies were identified by the MALDI-TOF MS method (Microflex LT Biotyper; Bruker Daltonics, Germany). Each WE sample was thus represented by zero to twenty isolates of *E. coli* (0–10 isolates resistant to cefotaxime and 0–10 with reduced susceptibility to ciprofloxacin), while from each G sample zero to two *E. coli* isolates (0–1 isolate resistant to cefotaxime, 0–1 with reduced susceptibility to ciprofloxacin) were obtained.

### 2.3 Antimicrobial susceptibility testing

All *E. coli* isolates were examined by disc diffusion test [12] for susceptibility to 16 antibacterial substances (Table S1). Double-disc synergy test [12] and D68C AmpC & ESBL Detection Set (Mast Diagnostics, UK) were used to evaluate the production of extended-spectrum beta-lactamases (ESBL) and AmpC type beta-lactamases.

### 2.4 Molecular typing of antibiotic-resistant *E. coli* isolates

The main criterion for the selection of isolates for further PCR-based typing was a match in the antibiotic resistance phenotypes between at least one WE and G isolate. Based on the profiles of phenotypic resistance, genes encoding selected beta-lactamases, genes for resistance to amphenicols, quinolones, streptomycin, sulphonamides and tetracyclines as well class 1 and 2 integrons were tested by PCR (Table S2). Sanger sequencing was applied to identify specific variants of *bla* and *qnr* genes*,* and the structure of variable regions of the integrons. PCR was used to assign *E. coli* isolates to phylogenetic groups [13] and for plasmid replicon typing [14].

### 2.5 Epidemiological relatedness of *E. coli* isolates

*E. coli* isolates from different sources with identical resistance profile and gene set were selected for further epidemiological typing. *Xba*I-restriction in combination with pulsed-field gel electrophoresis (PFGE) was performed according to standardised laboratory protocol [15]. Macrorestriction profiles were analysed with BioNumerics 6.6 fingerprinting software (Applied Maths, Belgium) and isolates were considered to be related if Dice similarity index was ≥85 % [16]. *E. coli* multi-locus sequence typing (MLST) was performed to assign sequence types (STs) (https://mlst.warwick.ac.uk/mlst/). MLST allelic profiles were used to construct a minimum spanning tree employing goeBURST full MLST algorithm as implemented in Phyloviz v2.0 package [17].

### 2.6 WGS, assembly, and data analysis of related *E. coli* isolates

The genomic DNA was extracted using NucleoSpin Tissue kit (Macherey-Nagel, Germany), and DNA libraries were prepared using Nextera XT DNA Library Preparation Kit (Illumina, Inc., USA) and subjected to sequencing on the MiSeq instrument (2 × 250 bp paired-end sequencing). Raw reads were quality trimmed (Trimmomatic v0.39) [18] and *de novo* assembled using SPAdes v3.12.0 [19]. The assemblies were screened for the resistance genes and chromosomal point mutations by ResFinder 3.1 [20], and for presence of plasmid replicons by PlasmidFinder 2.0 [21]. STs and phylogenetic groups of *E. coli* isolates were verified by MLST 2.0 [22] and ClermonTyping tools [23], respectively. ST131 subclones were identified based on the nucleotide sequence of *fimH* using FimTyper 1.0 [24] and variant of *bla*_CTX-M_ [25]. F-type plasmids were assigned to FAB formula using replicon sequence typing [26].

### 2.7 Comparative genomic analysis of selected isolates

Trimmed reads were mapped against *E. coli* str. K-12 substr. MG1655 (GenBank no. U00096.3) by bowtie2 v2.3.4.1 [27]. Single-nucleotide polymorphisms (SNPs) were detected separately for subsets of isolates belonging to ST131 or ST23, respectively, employing VarScan v2.3.9 [28]. All the sites in which at least one sample had read depth < 8 were discarded from further analysis. SNP distances among isolates were determined using Biostrings library [29] from the R environment v3.5.1. Phylogenetic tree of ST131 isolates based on detected SNP data was built by RAxML v8.2.10 [30] using the maximum-likelihood method. GTR model of nucleotide substitution with Γ rate of heterogeneity estimated from the data was identified as the most appropriate model for analysis through jModelTest v2.1.10 [31]. Robustness of the inferred tree topology was assessed by 500 bootstrap replicates. Final tree topology was visualised via iTOL v5.2 [32].

## 3 Results

### 3.1 Isolation and typing of antibiotic-resistant *E. coli* from wastewater effluents and gulls

A total of 670 *E. coli* isolates were obtained using selective cultivation. In case of WE (*n* = 450), at least one resistant isolate was obtained from each water sample and multi-resistance (resistance to three or more antibiotic substances) was detected in 66 % and 97 % WE isolates selected on MCA_cip_ (176/265) and MCA_cef_ (180/185), respectively. A total of 181 (64 %, *n* = 284) individual gulls were colonised by at least one antibiotic-resistant *E. coli* isolate and multi-resistance was demonstrated in 64 % (MCA_cef,_ 81/126) and 57 % (MCA_cip,_ 54/94) of G isolates. ESBL production was detected in 34 % isolates (229/670; 163 WE, 66 G) while 16 % of isolates (108/670; 55 WE, 53 G) produced AmpC type beta-lactamase. For further characterization, 146 isolates (76 WE, 70 G) with the same resistance phenotype were selected for genotyping. Results of phenotypic characterization are summarised in Table S1.

### 3.2 Detection of selected genetic markers in *E. coli* to compare WE and G isolates

From the total of 146 *E. coli* with the same resistance phenotype, 86 isolates (41 WE, 45 G) shared the same antibiotic resistance genes (Table S1) and this final set was selected for further genotyping (Fig. S1). Isolates were assigned to various phylogenetic groups with B2 being the most common (31 %, 19 WE, 8 G), followed by B1 (23 %) and A (21 %). Sequencing of the *bla* and *qnr* genes demonstrated the presence of *bla*_TEM-1_ (5 WE, 6 G), *bla*_CTX-M-1_ (2 WE, 3 G), *bla*_CTX-M-15_ (1 WE, 1 G)_,_ *bla*_CTX-M-27_ (9 WE, 2 G)*, bla*_CTX-M-174_ (3 WE)*, bla*_CMY-2_ (1 WE, 10 G) and *qnrS1* (1 WE, 1 G) variants. A class 1 integron 1.7 kb in size and containing a *dfr17-aadA5* gene cassette was found in six WE and three G isolates. Plasmid replicons were detected in 75 isolates (87 %, *n* = 86) with FIB (71 %) and FIA (24 %) being the most prevalent.

### 3.3 Phylogenetic relatedness of WE and G *E. coli*

**Fig. 1:**
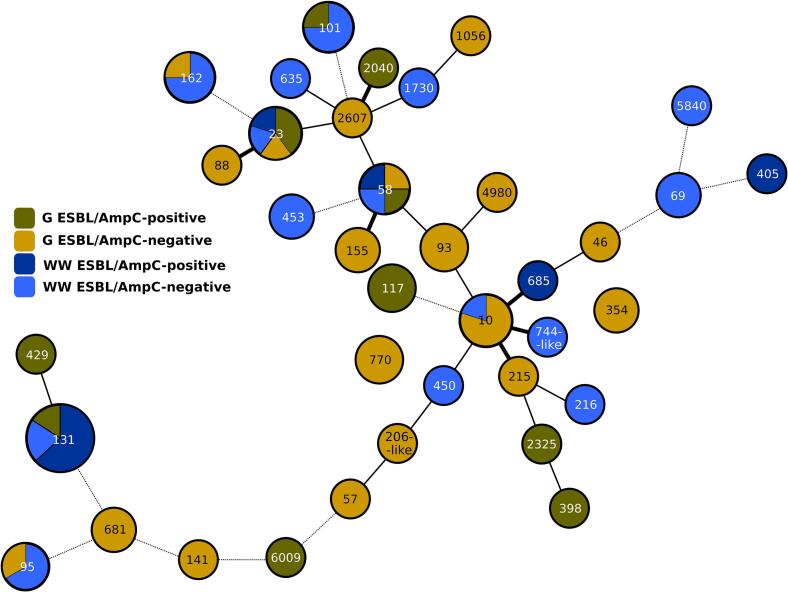
A minimum spanning tree of 85 *E. coli* isolates from wastewater effluents and Black-headed Gulls, based on MLST data. Each ST is represented by a separate circle. The size of the circle depends on the number of isolates. The thickness of the connecting line reflects a number of allelic differences between STs: thick line (1–2 differences), narrow line (3–4), dashed line (5–6). Strain origin: wastewater effluents (WW), gulls (G). One G isolate (R1350e) was not included into the analysis since it could not be assigned to a particular ST.

**Fig. 2:**
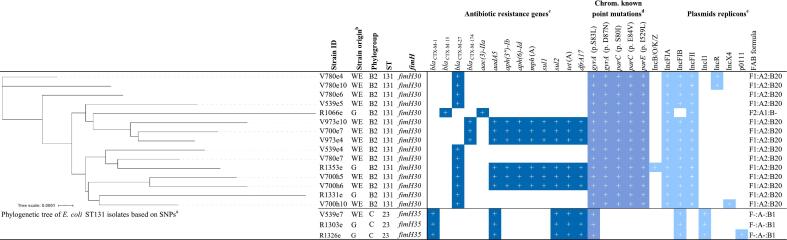
Characteristic of 18 *E. coli* isolates from wastewater effluents and gulls. ^a^Phylogenetic tree of *E. coli* ST23 isolates was conducted but not included due to negligible relatedness of the isolates (data not shown). ^b^Strain origin: wastewater effluents (WE), gulls (G). ^c^Antibiotic resistance genes: cut-off for positive detection was set up for at least 95 % identity and 100 % coverage to reference sequences. ^d^Chromosomal known point mutations conferring antimicrobial resistance. ^e^Plasmid replicons: cut-off for positive detection was set up for at least 95 % identity and coverage to reference sequences.

Thirty-seven different STs were identified among 86 isolates (Fig. S1, Fig. 1) with ST131 being the most common (16 WE, 3 G), followed by ST10 (1 WE, 4 G) and ST23 (2 WE, 3 G). High genetic diversity with 60 unique macrorestriction profiles among 86 isolates was demonstrated using PFGE (Fig. S1). Three subgroups (≥ 85 % similarity) of WE and G isolates belonging to ST131 (*n* = 15), ST23 (*n* = 3) and ST162 (n = 3) were identified (red highlighted in Fig. S1). Isolates of ST131 and ST23 were further typed by WGS (Fig. 2).

All ST131 isolates showed multi-resistance phenotype and carried genes conferring ESBL production. Majority of them (11/15) were classified as *H*30-R1 lineage carrying *bla*_CTX-M-27_ and F1:A2:B20 plasmid replicons*.* ST131 of WE origin (*n* = 12) were isolated from samples taken at different time points one month apart and all of which preceded the gulls sampling. ST23 included one wastewater isolate sampled two months prior to the two isolates originating from gulls. They were assigned to phylogenetic group C, contained *fimH35* and showed the same resistance phenotype, genotype and plasmid profile. ST162 (2 WE, 1 G) showed 88 % similarity of PFGE profiles, belonged to commensal phylogenetic group B1, were resistant to quinolones and carried FIB replicons. The WE isolates were obtained during two different samplings one month prior to the isolates from gulls.

Precise phylogenetic analysis of *E. coli* ST131 and ST23 was performed (Fig. 2). The number of SNPs detected in *E. coli* ST131 isolates ranged from 77 to 224 but the closest isolates from G and WE showed 90 SNPs difference. *E. coli* ST23 isolates showed the SNPs in range of 222–257. Due to the observed low relatedness among ST131 and ST23 isolates, the wastewater effluents cannot be concluded as a source of contamination of gull's nestlings in this study.

## 4 Discussion

The issue of antibiotic resistance in wastewaters has been previously addressed by a number of environmental studies pointing out the insufficient elimination of bacteria during treatment processes [[33], [34], [35]]. Here, the majority of *E. coli* isolated from effluents showed multi-resistance phenotype (79 %, 355/450), and 69 % WE isolates obtained on cefotaxime-supplemented medium (128/185) belonged to ESBL producers. The same municipal effluent was one-time sampled in 2016 followed by cultivation on cefotaxime-supplemented medium and 94 % of obtained *E. coli* (33/35) showed ESBL production [36]. It represents a high increase (from 69 % to 94 %) between the years 2012 and 2016.

In our study, we chose a Black-headed Gull to examine the transmission pathway of antibiotic-resistant enterobacteria into the environment via wastewater effluents. This bird species shows a synanthropic way of life together with a close link to the aquatic environment. Colonised birds can act as reservoirs of antibiotic-resistant bacteria including important human pathogens, therefore they play a significant role in the epidemiology of infectious diseases [[37], [38], [39], [40]].

A large study was conducted in gulls from nine European countries to test the north-south resistance gradient of antibiotic-resistant *E. coli* [41]. The worst situation was demonstrated in Spain where 61 % of gulls were colonised by *E. coli* resistant to at least one antibiotic even though antibiotic-supplemented agars were not used to select the isolates. The lowest prevalence of antibiotic-resistant *E. coli* was observed in gulls nesting in Denmark and Ireland, thus confirming that the north-south resistance gradient as observed in human medicine and livestock production may apply also to wildlife. In our study, the level of antibiotic-resistant *E. coli* was even higher (64 %) than in Spain, however, this result should be taken with caution as we used a different isolation method with antibiotic-supplemented agars. However, an isolation method with antibiotic-supplemented agars was used in another Swedish study [42] to evaluate gulls' colonisation by ESBL-producing *E. coli* in Malmö and Gothenburg with a final incidence of 17 %. High occurrence of antibiotic-resistant *E. coli* including ESBL producers in gulls in the Czech Republic has been reported previously using antibiotic-free media [43,44]. Up to 36 % of gulls from four different colonies located in northern and southern Moravia, Czech Republic, carried antibiotic-resistant *E. coli*. One of these four colonies was the same (Nove Mlyny colony) as in the present study and the occurrence of antibiotic-resistant *E. coli* in gulls was there 26 % in 2005 [43]. As antibiotic-supplemented media was used in the present study to select for resistant isolates, the lower carriage rate in 2005 can be explained by the different selection approach rather than the increasing prevalence of antibiotic resistance in gulls.

*E. coli* ST131 clone dominated and was identified in both sources. This clonal group is clinically important, globally disseminated and dominates in the aetiology of human extraintestinal coli-infections [45]. In addition to its ability to induce serious diseases, it commonly shows production of ESBL encoded by *bla*_CTX-M-15_ and *bla*_CTX-M-27_ and resistance to fluoroquinolones [46,47]. Interestingly, *bla*_CTX-M-27_ was found in eleven ST131-*H*30R1 isolates from both sources (WE, G) while only one isolate ST131-*H30*Rx with *bla*_CTX-M-15_ was identified. Of note, *bla*_CTX-M-27_-positive isolates belonging to the *H*30R1 clone have been isolated primarily from various clinical materials in a hospital located in the city of Brno [48]. Wastewaters from this hospital are treated by the same WWTP that was examined in our study. Other clinically important clonal lineages, namely ST69 and ST95, were detected in wastewater effluents and gulls in our study. These STs are frequently associated with neonatal meningitis, bloodstream and urinary tract infections in humans and have also been reported in various non-human sources such as wildlife, pets, or meat from retails [49].

The clonal relationship between enterobacteria isolated from wastewaters and water birds has recently been studied [7]. Extensive genetic relatedness through the PFGE and MLST methods was demonstrated on a set of quinolone-resistant *E. coli* isolated from clinical cases of human infections and from various environmental sources. In particular, isolates with similar genotypes originating from hospitalised patients, raw hospital wastewaters, urban surface water, treated wastewaters and gulls were identified. In another study from Bangladesh, *E. coli* isolates resistant to beta-lactams and belonging to the same STs commonly colonising humans and poultry were found in gull faeces [50]. We also observed a similar antibiotic profile, same STs and relatedness by PFGE of isolates originating from effluents and gulls, however the use of high-accurate WGS did not confirm the genetic identity across these two sources but rather showed some level of phylogenetic relatedness for some isolates.

## 5 Conclusion

Our work focused on the comparison of antibiotic-resistant *E. coli* isolates from wastewater effluents and gulls. We have shown that treated wastewaters discharged into the river Svratka contain high-risk multi-resistant *E. coli* clones despite the previous treatment process in the municipal WWTP. Our study also demonstrated a high carriage rate of antibiotic-resistant *E. coli* in Black-headed Gull nestlings in the colony downstream the river Svratka. In both sources, a high-risk ST131 clone was frequently detected, pointing to the probable circulation of clinical isolates in the environment.

In our collection of *E. coli*, we did not observe any closely related strains originating from these two sources thus we cannot conclude if contaminated river water can be a vehicle contributing to the colonisation of wild birds. We are aware of the limitation in the study design as a large preselection of molecularly typed isolates and the subsequent low number of sequenced isolates. Further work focused on other possible bacterial sources including feeding habits of gulls is required to clarify sources of contamination of the gulls by antibiotic-resistant bacteria.

The following are the supplementary data related to this article.

Table S1 Antibiotic resistance phenotypes and genotypes of 146 *E. coli* isolated from wastewater effluents and gulls. Table S1

Table S2 Listing of all tested genes and primers used in the study. Table S2

Supplementary material Figure S1. Characteristics of 86 *E. coli* isolates detected in wastewater effluents and Black-headed Gulls in the Czech Republic in 2012. Supplementary material

## Funding

The study was supported by the 10.13039/501100003243Ministry of Health of the Czech Republic (NU20J-09-00040 and DRO FNBr 65269705), the Internal Grant Agency of the University of Veterinary Sciences Brno, Czech Republic (grant No. 107/2017/FVL and 209/2023/FVHE). The study was also partially funded by projects 222/2024/FVHE and 209/2024/FVHE by University of Veterinary Sciences Brno, Czech Republic. Our thanks go to colleagues Jana Hofirkova and Jarmila Lausova for their laboratory work.

## CRediT authorship contribution statement

**Martina Masarikova:** Writing – review & editing, Writing – original draft, Methodology, Investigation, Funding acquisition, Conceptualization. **Iva Sukkar:** Writing – review & editing, Writing – original draft, Investigation, Data curation. **Ivana Jamborova:** Writing – review & editing, Writing – original draft, Methodology, Investigation. **Matej Medvecky:** Writing – review & editing, Writing – original draft, Visualization, Data curation. **Ivo Papousek:** Writing – review & editing, Writing – original draft, Investigation. **Ivan Literak:** Writing – review & editing, Writing – original draft, Supervision, Project administration, Conceptualization. **Alois Cizek:** Writing – review & editing, Writing – original draft, Supervision, Project administration, Conceptualization. **Monika Dolejska:** Writing – review & editing, Writing – original draft, Validation, Supervision, Resources, Project administration, Funding acquisition, Formal analysis, Conceptualization.

## Declaration of competing interest

The authors declare that they have no known competing financial interests or personal relationships that could have appeared to influence the work reported in this paper.

## Data Availability

The assemblies are available under BioProject PRJNA1007571, accession numbers SAMN37074019- SAMN37074036.

## References

[1] Rizzo L., Manaia C., Merlin C., Schwartz T., Dagot C., Ploy M.C., Michael I., Fatta-Kassinos D. (2013). Urban wastewater treatment plants as hotspots for antibiotic resistant bacteria and genes spread into the environment: a review. Sci. Total Environ..

[2] Uluseker C., Kaster K.M., Thorsen K., Basiry D., Shobana S., Jain M., Kumar G., Kommedal R., Pala-Ozkok I. (2021). A review on occurrence and spread of antibiotic resistance in wastewaters and in wastewater treatment plants: mechanisms and perspectives. Front. Microbiol..

[3] Berglund B. (2015). Environmental dissemination of antibiotic resistance genes and correlation to anthropogenic contamination with antibiotics. Infect Ecol. Epidemiol..

[4] Bouki C., Venieri D., Diamadopoulos E. (2013). Detection and fate of antibiotic resistant bacteria in wastewater treatment plants: a review. Ecotoxicol. Environ. Saf..

[5] Quintela-Baluja M., Abouelnaga M., Romalde J., Su J.Q., Yu Y., Gomez-Lopez M., Smets B., Zhu Y.G., Graham D.W. (2019). Spatial ecology of a wastewater network defines the antibiotic resistance genes in downstream receiving waters. Water Res..

[6] Herrig I., Fleischmann S., Regnery J., Wesp J., Reifferscheid G., Manz W. (2020). Prevalence and seasonal dynamics of *bla*_CTX-M_ antibiotic resistance genes and fecal indicator organisms in the lower Lahn River, Germany. PLoS One.

[7] Varela A.R., Manageiro V., Ferreira E., Guimarães M.A., Da Costa P.M., Caniça M., Manaia C.M. (2015). Molecular evidence of the close relatedness of clinical, gull and wastewater isolates of quinolone-resistant *Escherichia coli*. J. Glob. Antimicrob. Resist..

[8] Wang J., Ma Z.B., Zeng Z.L., Yang X.W., Huang Y., Liu J.H. (2017). The role of wildlife (wild birds) in the global transmission of antimicrobial resistance genes. Zool. Res..

[9] Smith G.C., Carlile N. (1993). Methods for population control within a silver Gull Colony. Wildl. Res..

[10] Masarikova M., Manga I., Cizek A., Dolejska M., Oravcova V., Myskova P., Karpiskova R., Literak I. (2016). *Salmonella enterica* resistant to antimicrobials in wastewater effluents and black-headed gulls in the Czech Republic, 2012. Sci. Total Environ..

[11] Oravcova V., Mihalcin M., Zakova J., Pospisilova L., Masarikova M., Literak I. (2017). Vancomycin-resistant enterococci with *vanA* gene in treated municipal wastewater and their association with human hospital strains. Sci. Total Environ..

[12] CLSI, Clinical and Laboratory Standards Institute (2015).

[13] Clermont O., Christenson J.K., Denamur E., Gordon D.M. (2013). The Clermont *Escherichia coli* phylo-typing method revisited: improvement of specificity and detection of new phylo-groups. Environ. Microbiol. Rep..

[14] Carattoli A., Bertini A., Villa L., Falbo V., Hopkins K.L., Threlfall E.J. (2005). Identification of plasmids by PCR-based replicon typing. J. Microbiol. Methods.

[15] Centers for Disease Control and Prevention (2004).

[16] Carriço J.A., Pinto F.R., Simas C., Nunes S., Sousa N.G., Frazão N., De Lencastre H., Almeida J.S. (2005). Assessment of band-based similarity coefficients for automatic type and subtype classification of microbial isolates analyzed by pulsed-field gel electrophoresis. J. Clin. Microbiol..

[17] Nascimento M., Sousa A., Ramirez M., Francisco A.P., Carriço J.A., Vaz C. (2017). PHYLOViZ 2.0: providing scalable data integration and visualization for multiple phylogenetic inference methods. Bioinformatics.

[18] Bolger A.M., Lohse M., Usadel B. (2014). Trimmomatic: a flexible trimmer for Illumina sequence data. Bioinformatics.

[19] Bankevich A., Nurk S., Antipov D., Gurevich A.A., Dvorkin M., Kulikov A.S., Lesin V.M., Nikolenko S.I., Pham S., Prjibelski A.D., Pyshkin A.V., Sirotkin A.V., Vyahhi N., Tesler G., Alekseyev M.A., Pevzner P.A. (2012). SPAdes: a new genome assembly algorithm and its applications to single-cell sequencing. J. Comput. Biol..

[20] Zankari E., Hasman H., Cosentino S., Vestergaard M., Rasmussen S., Lund O., Aarestrup F.M., Larsen M.V. (2012). Identification of acquired antimicrobial resistance genes. J. Antimicrob. Chemother..

[21] Carattoli A., Zankari E., Garciá-Fernández A., Larsen M.V., Lund O., Villa L., Aarestrup F.M., Hasman H. (2014). *In silico* detection and typing of plasmids using plasmidfinder and plasmid multilocus sequence typing. Antimicrob. Agents Chemother..

[22] Larsen M.V., Cosentino S., Rasmussen S., Friis C., Hasman H., Marvig R.L., Jelsbak L., Sicheritz-Pontén T., Ussery D.W., Aarestrup F.M., Lund O. (2012). Multilocus sequence typing of total-genome-sequenced bacteria. J. Clin. Microbiol..

[23] Beghain J., Bridier-Nahmias A., Le Nagard H., Denamur E., Clermont O. (2018). ClermonTyping: an easy-to-use and accurate *in silico* method for *Escherichia* genus strain phylotyping. Microb. Genom..

[24] Roer L., Tchesnokova V., Allesøe R., Muradova M., Chattopadhyay S., Ahrenfeldt J., Thomsen M.C.F., Lund O., Hansen F., Hammerum A.M., Sokurenko E., Hasman H. (2017). Development of a web tool for *Escherichia coli* subtyping based on *fimH* alleles. J. Clin. Microbiol..

[25] Johnson J.R., Porter S., Thuras P., Castanheira M. (2017). The pandemic *H30* subclone of sequence type 131 (ST131) as the leading cause of multidrug-resistant *Escherichia coli* infections in the United States (2011−2012). Open Forum Infect. Dis..

[26] Villa L., García-Fernández A., Fortini D., Carattoli A. (2010). Replicon sequence typing of IncF plasmids carrying virulence and resistance determinants. J. Antimicrob. Chemother..

[27] Langmead B., Salzberg S.L. (2012). Fast gapped-read alignment with bowtie 2. Nat. Methods.

[28] Koboldt D.C., Zhang Q., Larson D.E., Shen D., McLellan M.D., Lin L., Miller C.A., Mardis E.R., Ding L., Wilson R.K. (2012). VarScan 2: somatic mutation and copy number alteration discovery in cancer by exome sequencing. Genome Res..

[29] Pages H., Aboyoun P., Gentleman R., Debroy S. (2019). Biostrings: Efficient Manipulation of Biological Strings. R Package Version 2.50.2. https://bioconductor.org/packages/release/bioc/html/Biostrings.html.

[30] Stamatakis A. (2014). RAxML version 8: a tool for phylogenetic analysis and post-analysis of large phylogenies. Bioinformatics.

[31] Darriba D., Taboada G.L., Doallo R., Posada D. (2012). JModelTest 2: more models, new heuristics and parallel computing. Nat. Methods.

[32] Letunic I., Bork P. (2019). Interactive tree of life (iTOL) v4: recent updates and new developments. Nucleic Acids Res..

[33] Caltagirone M., Nucleo E., Spalla M., Zara F., Novazzi F., Marchetti V.M., Piazza A., Bitar I., De Cicco M., Paolucci S., Pilla G., Migliavacca R., Pagani L. (2017). Occurrence of extended spectrum β-lactamases, KPC-type, and MCR-1.2-producing *Enterobacteriaceae* from wells, river water, and wastewater treatment plants in Oltrepò Pavese area, northern Italy. Front. Microbiol..

[34] Dolejska M., Frolkova P., Florek M., Jamborova I., Purgertova M., Kutilova I., Cizek A., Guenther S., Literak I. (2011). CTX-M-15-producing *Escherichia coli* clone B2-O25b-ST131 and *Klebsiella* spp. isolates in municipal wastewater treatment plant effluents. J. Antimicrob. Chemother..

[35] Verburg I., García-Cobos S., Leal L.H., Waar K., Friedrich A.W., Schmitt H. (2019). Abundance and antimicrobial resistance of three bacterial species along a complete wastewater pathway. Microorganisms.

[36] Kutilova I., Medvecky M., Leekitcharoenphon P., Munk P., Masarikova M., Davidova-Gerzova L., Jamborova I., Bortolaia V., Pamp S.J., Dolejska M. (2021). Extended-spectrum beta-lactamase-producing *Escherichia coli* and antimicrobial resistance in municipal and hospital wastewaters in Czech Republic: culture-based and metagenomic approaches. Environ. Res..

[37] Dolejska M., Masarikova M., Dobiasova H., Jamborova I., Karpiskova R., Havlicek M., Carlile N., Priddel D., Cizek A., Literak I. (2016). High prevalence of *Salmonella* and IMP-4-producing Enterobacteriaceae in the silver gull on five islands, Australia. J. Antimicrob. Chemother..

[38] Literak I., Dolejska M., Janoszowska D., Hrusakova J., Meissner W., Rzyska H., Bzoma S., Cizek A. (2010). Antibiotic-resistant *Escherichia coli* bacteria, including strains with genes encoding the extended-spectrum beta-lactamase and QnrS, in waterbirds on the Baltic Sea coast of Poland. Appl. Environ. Microbiol..

[39] Literak I., Manga I., Wojczulanis-Jakubas K., Chroma M., Jamborova I., Dobiasova H., Sedlakova M.H., Cizek A. (2014). *Enterobacter cloacae* with a novel variant of ACT AmpC beta-lactamase originating from glaucous gull (*Larus hyperboreus*) in Svalbard. Vet. Microbiol..

[40] Vittecoq M., Godreuil S., Prugnolle F., Durand P., Brazier L., Renaud N., Arnal A., Aberkane S., Jean-Pierre H., Gauthier-Clerc M., Thomas F., Renaud F. (2016). REVIEW: antimicrobial resistance in wildlife. J. Appl. Ecol..

[41] Stedt J., Bonnedahl J., Hernandez J., McMahon B.J., Hasan B., Olsen B., Drobni M., Waldenström J. (2014). Antibiotic resistance patterns in *Escherichia coli* from gulls in nine European countries. Infect Ecol. Epidemiol..

[42] Atterby C., Börjesson S., Ny S., Järhult J.D., Byfors S., Bonnedahl J. (2017). ESBL-producing *Escherichia coli* in Swedish gulls—a case of environmental pollution from humans?. PLoS One.

[43] Dolejska M., Cizek A., Literak I. (2007). High prevalence of antimicrobial-resistant genes and integrons in *Escherichia coli* isolates from black-headed gulls in the Czech Republic. J. Appl. Microbiol..

[44] Dolejska M., Bierosova B., Kohoutova L., Literak I., Cizek A. (2009). Antibiotic-resistant *Salmonella* and *Escherichia coli* isolates with integrons and extended-spectrum beta-lactamases in surface water and sympatric black-headed gulls. J. Appl. Microbiol..

[45] Nicolas-Chanoine M.H., Bertrand X., Madec J.Y. (2014). *Escherichia coli* ST131, an intriguing clonal group. Clin. Microbiol. Rev..

[46] Banerjee R., Johnson J.R. (2014). A new clone sweeps clean: the enigmatic emergence of *Escherichia coli* sequence type 131. Antimicrob. Agents Chemother..

[47] Mathers A.J., Peirano G., Pitout J.D.D. (2015). The role of epidemic resistance plasmids and international high- risk clones in the spread of multidrug-resistant *Enterobacteriaceae*. Clin. Microbiol. Rev..

[48] Jamborova I., Johnston B.D., Papousek I., Kachlikova K., Micenkova L., Clabots C., Skalova A., Chudejova K., Dolejska M., Literak I., Johnson J.R. (2018). Extensive genetic commonality among wildlife, wastewater, community, and nosocomial isolates of *Escherichia coli* sequence type 131 (*H*30R1 and *H*30Rx subclones) that carry *bla*_CTX-M-27_ or *bla*_CTX-M-15_. Antimicrob. Agents Chemother..

[49] Riley L.W. (2014). Pandemic lineages of extraintestinal pathogenic *Escherichia coli*. Clin. Microbiol. Infect..

[50] Hasan B., Melhus Å., Sandegren L., Alam M., Olsen B. (2014). The gull (*Chroicocephalus brunnicephalus*) as an environmental bioindicator and reservoir for antibiotic resistance on the coastlines of the bay of Bengal. Microb. Drug Resist..

