# Supplementary material for: Antibiotic-resistant Escherichia coli from treated municipal wastewaters and Black-headed Gull nestlings on the recipient river
Source: One Health. 2024 Sep 22;19:100901. doi: 10.1016/j.onehlt.2024.100901 (PMC11470789; doi:10.1016/j.onehlt.2024.100901)
Supplement: Table S1 — Antibiotic resistance phenotypes and genotypes of 146 E. coli isolated from wastewater effluents and gulls. [file mmc1.docx]

**Table S1. Antibiotic resistance phenotypes and genotypes of 146 *E. coli* isolated from wastewaters effluents and gulls.**

| **ID** | **Samp** | **I** | **Resistance phenotype** | **Resistance genotype** |
| --- | --- | --- | --- | --- |
| V415h8 | WE | cip | ACfCipCtxNaSSuSxtT | *bla*_TEM_, *bla*_CTX-M_, *strA*, *sul2*, *tetB* |
| V539h2 | WE | cip | ACipGnNaSSuSxtT | *bla*_TEM-1_, *strA*, *sul1*, *sul2, tetA*; *intI1,* class 1 integron |
| V539h7 | WE | cip | ACfCipCtxNaSSuSxtT | *strA, sul1, sul2*, *tetA* |
| V700h5 | WE | cip | ACfCipCtxNaSSuSxtT | *bla*_CTX-M-27_, *strA*, *sul1*, *sul2, tetA*; *intI1,* class 1 integron |
| V700h6 | WE | cip | ACfCipCtxNaSSuSxtT | *bla*_CTX-M-27_*, strA, sul1, sul2, tetA* |
| V970h4 | WE | cip | ACCipNaSSuSxtT | *bla*_TEM-1_, *cat*, *strA*, *sul1*, *sul2, tetB*; *intI1,* class 1 integron |
| V970h5 | WE | cip | ACCipNaSSuSxtT | *bla*_TEM_*, catA, strA, sul2,* *tetB* |
| R1294h | G | cip | ACipGnNaSSuSxtT | *bla*_TEM-1_, *strA*, *sul1*, *sul2, tetA*; *intI1,* class 1 integron |
| V539e7 | WE | cef | ACfCtxNaSuSxtT | *bla*_CTX-M-1_, *sul2*, *tetA* |
| V700e7 | WE | cef | ACfCipCtxNaSSuSxtT | *bla*_CTX-M-174_, *strA*, *sul1*, *sul2, tetA; intI1,* class 1 integron |
| V782e1 | WE | cef | ACfCipCtxNaSSuSxtT | *bla*_CTX-M_, *sul1,* *tetA* |
| V973e2 | WE | cef | ACfCipCtxNaSSuSxtT | *bla*_CTX-M,_ *strA, sul2,* *tetA* |
| V973e4 | WE | cef | ACfCipCtxNaSSuSxtT | *bla*_CTX-M-174_*, strA, sul1, sul2, tetA; intI1,* class 1 integron |
| V973e10 | WE | cef | ACfCipCtxNaSSuSxtT | *bla*_CTX-M-174_, *strA*, *sul1*, *sul2, tetA*; *intI1,* class 1 integron |
| R1132e | G | cef | ACfCtxNaSuSxtT | *bla*_CTX-M_, *sul2,* *tetA* |
| R1133e | G | cef | ACfCtxNaSuSxtT | *bla*_CTX-M_, *sul2,* *tetA* |
| R1213e | G | cef | ACfCtxNaSuSxtT | *bla*_CTX-M_, *sul2*, *tetA* |
| R1303e | G | cef | ACfCtxNaSuSxtT | *bla*_CTX-M-1_, *sul2*, *tetA* |
| R1305e | G | cef | ACfCtxNaSSxtT | *bla*_CTX-M_, *sul2*, *tetA* |
| R1326e | G | cef | ACfCtxNaSuSxtT | *bla*_CTX-M-1_, *sul2*, *tetA* |
| R1342e | G | cef | ACfCtxNaSuSxtT | *bla*_CTX-M_, *sul2* |
| R1353e | G | cef | ACfCipCtxNaSSuSxtT | *bla*_CTX-M-27_*, strA, sul1, sul2, tetA*; *intI1,* class 1 integron |
| V279e1 | WE | cef | ACfCtxSuSxt | *bla*_CTX-M_, *sul1, sul2* |
| V279e3 | WE | cef | ACfCtxSuSxt | *bla*_CTX-M_, *sul1, sul2* |
| V415e1 | WE | cef | ACfCtxSuSxt | *bla*_TEM_, *bla*_CTX-M_, *qnrB, sul1*, *tetB* |
| V415e2 | WE | cef | ACfCtxSuSxt | *bla*_TEM_, *bla*_CTX-M_, *qnrB, sul1,* *tetB* |
| V539e6 | WE | cef | AAcCazCfCtxFox | ­- |
| V780e2 | WE | cef | ACazCfCipCtxNa | *bla*_CTX-M-15_ |
| V1047e3 | WE | cef | AAcCfFoxNaT | *bla*_TEM_, *tetB* |
| R1066e | G | cef | ACazCfCipCtxNa | *bla*_CTX-M-15_ |
| R1270e | G | cef | AAcCazCfCtxFox | ­- |
| R1340e | G | cef | AAcCfFox | ­- |
| R1345e | G | cef | ACfCtxSuSxt | *bla*_CTX-M_, *sul2* |
| V279h1 | WE | cip | ANaSSuSxtT | *bla*_TEM_, *strA, sul1,* *tetA* |
| V279h5 | WE | cip | ANaSSuSxtT | *bla*_TEM_, *strA, sul2,* *tetB* |
| V415h1 | WE | cip | ANaSSuSxt | *bla*_TEM-1_, *strA*, *sul2* |
| V415h3 | WE | cip | ACfCipNaT | *tetA* |
| V539h10 | WE | cip | ANaSSuSxtT | *strA, sul2,* *tetB* |
| V700h3 | WE | cip | ACipNaSu | *sul2* |
| V782h7 | WE | cip | ANaSSuSxtT | *bla*_TEM_, *strA, sul1, sul2*, *tetA*, *tetB* |
| V782h9 | WE | cip | ANaSSuSxtT | *bla*_TEM_, *strA, sul2,* *tetA* |
| R1071h | G | cip | ANaSSuSxt | *bla*_TEM-1_, *strA*, *sul2* |
| R1103h | G | cip | ANaSSuSxtT | *bla*_TEM_, *strA, sul1, sul2,* *tetA* |
| R1276h | G | cip | ACfCipNaT | *bla*_TEM_, *qnrS*, *tetA*, *tetB* |
| V539h8 | WE | cip | ACfCipCtxNa | *bla*_TEM_, *bla*_CTX-M_ |
| V700h10 | WE | cip | ACfCipCtxNa | *bla*_CTX-M-27_ |
| V279e8 | WE | cef | ACfCipCtxNa | *bla*_TEM_, *bla*_CTX-M_ |
| V539e4 | WE | cef | ACfCipCtxNa | *bla*_CTX-M-27_ |
| V539e5 | WE | cef | ACfCipCtxNa | *bla*_CTX-M-27_ |
| V780e4 | WE | cef | ACfCipCtxNa | *bla*_CTX-M-27_ |
| V780e6 | WE | cef | ACfCipCtxNa | *bla*_CTX-M-27_ |
| V780e7 | WE | cef | ACfCipCtxNa | *bla*_CTX-M-27_ |
| V780e10 | WE | cef | ACfCipCtxNa | *bla*_CTX-M-27_ |
| R1331e | G | cef | ACfCipCtxNa | *bla*_CTX-M-27_ |
| V700h2 | WE | cip | ACipNaSu | *sul2* |
| V700h4 | WE | cip | ACipNaSu | *sul2* |
| V700h8 | WE | cip | ACipNaSu | *sul2* |
| V415h5 | WE | cip | ANaSuT | *bla*_TEM_, *sul2*, *tetB* |
| V973h9 | WE | cip | ANaSuT | *bla*_TEM_, *sul2*, *tetA* |
| R1091h | G | cip | ACipNaT | *bla*_TEM_, *tetA* |
| R1234h | G | cip | ACipNaT | *bla*_TEM_, *sul1*, *tetA* |
| R1255h | G | cip | ACipNaT | *tetA* |
| V970h9 | WE | cip | ACipNaT | *bla*_TEM_, *tetB* |
| R1123h | G | cip | ACipNaSu | *bla*_TEM_, *sul2* |
| R1245h | G | cip | ANaSuT | *sul2*, *tetA* |
| V700e4 | WE | cef | ACfCipCtxNa | ­- |
| R1197e | G | cef | AAcCfFox | ­- |
| R1060e | G | cef | AAcCfFox | *bla*_CMY-2_ |
| R1142e | G | cef | AAcCfFox | *bla*_CMY-2_ |
| R1170e | G | cef | AAcCfFox | *bla*_CMY-2_ |
| R1185e | G | cef | AAcCfFox | *bla*_CMY-2_ |
| R1200e | G | cef | AAcCfFox | *bla*_CMY-2_ |
| R1214e | G | cef | AAcCfFox | *bla*_CMY-2_ |
| V415e3 | WE | cef | AAcCfFox | *bla*_CMY-2_ |
| R1219e | G | cef | AAcCfFox | *bla*_CMY-2_ |
| R1237e | G | cef | AAcCfFox | ­- |
| R1272e | G | cef | AAcCfFox | *bla*_CMY-2_ |
| V539e8 | WE | cef | ACfCtx | *bla*_CTX-M-1_ |
| V780e9 | WE | cef | ACfCtx | *bla*_CTX-M_, *qnrS* |
| R1149e | G | cef | AAcCfFoxNaT | *bla*_TEM_, *bla*_CMY_, *tetB* |
| R1307e | G | cef | AAcCfFox | *bla*_CMY-2_ |
| R1317e | G | cef | AAcCfFox | *bla*_CMY-2_ |
| R1175e | G | cef | ACfCtx | *bla*_SHV_, *qnrS* |
| R1348e | G | cef | ACfCtx | *bla*_CTX-M_ |
| R1351e | G | cef | ACfCtx | *bla*_CTX-M_ |
| R1319e | G | cef | ACfCtx | *bla*_TEM_, *bla*_CTX-M_ |
| R1350e | G | cef | ACfCtx | *bla*_CTX-M-1_ |
| R1216h | G | cip | CipNa | ­- |
| V973h7 | WE | cip | ANaT | *tetA*, *tetB* |
| V970h10 | WE | cip | CipNa | ­- |
| V1052h6 | WE | cip | CipNa | ­- |
| R1136h | G | cip | ANaT | *bla*_TEM_, *qnrB*, *tetA*, *tetB* |
| R1272h | G | cip | ANaT | *bla*_TEM_, *tetB* |
| R1275h | G | cip | ANaT | *bla*_TEM_, *tetB* |
| R1297h | G | cip | CipNa | ­- |
| V279h8 | WE | cip | CipNa | ­- |
| V415h4 | WE | cip | CipNa | ­- |
| V780h8 | WE | cip | CipNa | ­- |
| V780h10 | WE | cip | CipNa | ­- |
| V782h1 | WE | cip | ACipNaSSuSxt | *bla*_TEM_, *strA, sul2,* *tetB* |
| V782h2 | WE | cip | CipNa | ­- |
| V970h1 | WE | cip | CipNa | ­- |
| V1008h10 | WE | cip | Na | ­- |
| V1052h1 | WE | cip | Na | ­- |
| V1052h8 | WE | cip | Na | *aac(6´)-Ib*, *tetB* |
| V1052h5 | WE | cip | CipNa | ­- |
| R1216h | G | cip | CipNa | ­- |
| R1297h | G | cip | CipNa | ­- |
| V1052e10 | WE | cef | Na | ­- |
| V279h2 | WE | cip | Na | ­- |
| V539h6 | WE | cip | Na | *qnrS*, *tetB* |
| V700h1 | WE | cip | Na | ­- |
| V700h7 | WE | cip | Na | ­- |
| V780h3 | WE | cip | Na | ­- |
| R1068h | G | cip | Na | ­- |
| R1070h | G | cip | Na | ­- |
| R1099h | G | cip | CipNa | ­- |
| R1073h | G | cip | Na | ­- |
| R1090h | G | cip | Na | ­- |
| R1157h | G | cip | Na | ­- |
| R1132h | G | cip | Na | ­- |
| R1147h | G | cip | Na | ­- |
| R1222h | G | cip | Na | ­- |
| R1230h | G | cip | Na | ­- |
| R1247h | G | cip | Na | ­- |
| R1281h | G | cip | Na | ­- |
| R1304h | G | cip | Na | ­- |
| R1311h | G | cip | Na | ­- |
| R1324h | G | cip | Na | ­- |
| R1240h | G | cip | ANa | *bla*_TEM-1_ |
| R1062h | G | cip | ACipNaSSuSxt | *sul1, sul2,* *tetB* |
| V279h4 | WE | cip | Na | ­- |
| V782h3 | WE | cip | Na | *strA*, *sul2*, *tetB* |
| V782h10 | WE | cip | Na | ­- |
| R1102h | G | cip | CipNa | ­- |
| R1156h | G | cip | CipNa | ­- |
| R1310h | G | cip | ANa | *bla*_TEM-1_ |
| R1067h | G | cip | ANa | *bla*_TEM-1_ |
| V539e1 | WE | cef | ACfCtx | *bla*_CTX-M_, *tetB* |
| V539e2 | WE | cef | ACfCtx | *bla*_CTX-M_, *tetB* |
| V782h4 | WE | cip | ANa | *bla*_TEM-1_ |
| V782h6 | WE | cip | ANa | *bla*_TEM-1_ |
| R1228h | G | cip | ND | *qnrS1* |
| R1279h | G | cip | ACCipNaSSuSxtT | *bla*_TEM-1_, *cat*, *strA*, *sul1*, *sul2, tetB*; *intI1,* class 1 integron |
| V700e3 | WE | cef | ACfCipCtxNa | *bla*_CTX-M_, *strA*, *sul2*, *tetA*, *tetB* |
| V539h1 | WE | cip | ND | *qnrS1* |

**Notes:**

No, Number of the isolate; ID, Identification number of the isolate; Samp, Sample; I, Isolation on MCA agar with cefotaxime (cef) or MCA with ciprofloxacin (cip). WE, wastewater effluent; G, gull;

Eighty-six isolates selected for final molecular characterization are highlighted red.

Resistance phenotype: ampicillin (A, 10 μg), amoxicillin-clavulanic acid (Ac, 20/10 μg), cephalothin (Cf, 30 μg), cefoxitin (Fox, 30 μg), ceftazidime (Caz, 30 μg), cefotaxime (Ctx, 30 μg), chloramphenicol (C, 30 μg), nalidixic acid (Na, 30 μg), ciprofloxacin (Cip, 5 μg), streptomycin (S, 10 μg), gentamicin (Gn, 10 μg), sulphonamide compounds (Su, 300 μg), sulphamethoxazole-trimethoprim (Sxt, 23,75/1,25 μg), tetracycline (T, 30 μg), imipenem (Ipm, 10 μg) and meropenem (Mem,10 μg).

All class 1 integrons are 1.7 kb in size and contain a *dfr17-aadA5* gene cassettes.
