# Supplementary material for: Antibiotic-resistant Escherichia coli from treated municipal wastewaters and Black-headed Gull nestlings on the recipient river
Source: One Health. 2024 Sep 22;19:100901. doi: 10.1016/j.onehlt.2024.100901 (PMC11470789; doi:10.1016/j.onehlt.2024.100901)
Supplement: Table S2 — Listing of all tested genes and primers used in the study. [file mmc2.docx]

**Table S2. Listing of all tested genes and primers used in the study.**

| **Target** | **Gene** | **Primer** | **Primer sequence (5'-3')** | **Reference/ GenBank** | **Note** |
| --- | --- | --- | --- | --- | --- |
|  |  |  |  |  |  |
| **ESBL** | *bla*_CTX-M_ | PANCTX-M-F | TTT GCG ATG TGC AGT ACC AGT AA | Lewis *et al.* 2007 | PCR screening for *bla*_CTX-M_ genes |
|  |  | PANCTX-M-R | CGA TAT CGT TGG TGG TGC CAT A |  |  |
|  | *bla*_CTX-M group I_ | CTXM1-F3 | GAC GAT GTC ACT GGC TGA GC | Pitout *et al.* 2004 | PCR screening for *bla*_CTX-M_ group I genes |
|  |  | CTXM1-R2 | AGC CGC CGA CGC TAA TAC A |  |  |
|  | *bla*_CTX-M group II_ | TOHO1-2F | GCG ACC TGG TTA ACT ACA ATC C | Pitout *et al.* 2004 | PCR screening for *bla*_CTX-M_ group II genes |
|  |  | TOHO1-1R | CGG TAG TAT TGC CCT TAA GCC |  |  |
|  | *bla*_CTX-M group III_ | CTXM825F | CGC TTT GCC ATG TGC AGC ACC | Pitout *et al.* 2004 | PCR screening for *bla*_CTX-M_ group III genes |
|  |  | CTXM825R | GCT CAG TAC GAT CGA GCC |  |  |
|  | *bla*_CTX-M group IV_ | CTXM914-F | GCT GGA GAA AAG CAG CGG AG | Pitout *et al.* 2004 | PCR screening for *bla*_CTX-M_ group IV genes |
|  |  | CTXM914-R | GTA AGC TGA CGC AAC GTC TG |  |  |
|  | *bla*_CTX-M group V_ | CTX-M- group 5-F | CGC CGA TAA CAC GCA GAC | McGettigan *et al.* 2009 | PCR screening for *bla*_CTX-M_ group V genes |
|  |  | CTX-M- group 5-R | CGG CTC CGA CTG GGT GAA GTA |  |  |
|  | *bla*_CTX-M group I_^seq^ | CTX-M-1seq-F | TCT TCC AGA ATA AGG AAT CCC | Sturenburg *et al.* 2004 | PCR for Sanger sequencing |
|  |  | CTX-M-1seq-R | CCG TTT CCG CTA TTA CAA AC |  |  |
|  | *bla*_CTX-M group II_^seq^ | CTX-M-gr.2 seg R | ATG ATG ACT CAG AGC ATT CGC | X92507 | PCR for Sanger sequencing |
|  |  | CTX-M-gr.2 seg F | TCG CTC CAT TTA TTG CAT CA |  |  |
|  | *bla*_CTX-M group IV_^seq^ | CTX-M-9-like-F | ATG GTG ACA AAG AGA GTG CAA CG | AF174129 | PCR for Sanger sequencing |
|  |  | CTX-M-9-like-R | TTA CAG CCC TTC GGC GAT GAT |  |  |
| **AmpC beta-lactamases** | *bla*_MOX_*, bla*_CMY-1 group_ | MOXMF | GCT GCT CAA GGA GCA CAG GAT | Perez-Perez *et al.* 2002 | PCR screening |
|  |  | MOXMR | CAC ATT GAC ATA GGT GTG GTG C |  |  |
|  | *bla*_CMY-2 group,_ *bla*_LAT_ | CITMF | TGG CCA GAA CTG ACA GGC AAA | Perez-Perez *et al.* 2002 | PCR screening |
|  |  | CITMR | TTT CTC CTG AAC GTG GCT GGC |  |  |
|  | *bla*_DHA_ | DHAF | AAC TTT CAC AGG TGT GCT GGG T | Perez-Perez *et al.* 2002 | PCR screening |
|  |  | DHAR | CCG TAC GCA TAC TGG CTT TGC |  |  |
|  | *bla*_AAC_ | AACMF | AAC AGC CTC AGC AGC CGG TTA | Perez-Perez *et al.* 2002 | PCR screening |
|  |  | AACMR | TTC GCC GCA ATC ATC CCT AGC |  |  |
|  | *bla*_MIR-1_, *bla*_ACT-1_ | EBCMF | CTT CCA CTG CGG CTG CCA GTT | Perez-Perez *et al.* 2002 | PCR screening |
|  |  | EBCMR | TCG GTA AAG CCG ATG TTG CGG |  |  |
|  | *bla*_FOX_ | FOXMF | AAC ATG GGG TAT CAG GGA GAT G | Perez-Perez *et al.* 2002 | PCR screening |
|  |  | FOXMR | CAA AGC GCG TAA CCG GAT TGG |  |  |
|  | *bla*_CMY-2_^seq^ | blaCMYseq-F | AAC ACA CTG ATT GCG TCT | Agerso *et al.* 2012 | PCR for Sanger sequencing |
|  |  | blaCMYseq-R | CTG GGC CTC ATC GTC AGT |  |  |
| **Other beta-lactamases** | *bla*_OXA-1 group_ | blaOXA-F | TTC AAG CCA AAG GCA CGA TAG | Brinas *et al.* 2002 | PCR screening |
|  |  | blaOXA-R | TCC GAG TTG ACT GCC GGG TTG |  |  |
|  | *bla*_SHV_ | blaSHV-F | CAC TCA AGG ATG TAT TGT G |  | PCR screening |
|  |  | blaSHV-R | TTA GCG TTG CCA GTG CTC G |  |  |
|  | *bla*_TEM_ | blaTEM-F | TT CTT GAA GAC GAA AGG GC |  | PCR screening |
|  |  | blaTEM-R | ACG CTC AGT GGA ACG AAA AC |  |  |
| **Plasmid-mediated quinolone resistance** | *qnrA* | qnrA-F | ATT TCT CAC GCC AGG ATT TG | Robicsek *et al.* 2006 | PCR screening/PCR for Sanger sequencing |
|  |  | qnrA-R | GAT CGG CAA AGG TTA GGT CA |  |  |
|  | *qnrB* | qnrB-F | GAT CGT GAA AGC CAG AAA GG | Bin *et al.* 2009 | PCR screening |
|  |  | qnrB-R | ATG AGC AAC GAT GCC TGG TA |  |  |
|  | *qnrC* | qnrC-F | GGG TTG TAC ATT TAT TGA ATC G | Bin *et al.* 2009 | PCR screening/PCR for Sanger sequencing |
|  |  | qnrC-R | CAC CTA CCC ATT TAT TTT CA |  |  |
|  | *qnrD* | qnrD-F | CGA GAT CAA TTT ACG GGG AAT A | Cavaco *et al.* 2009 | PCR screening/PCR for Sanger sequencing |
|  |  | qnrD-R | AAC AAG CTG AAG CGC CTG |  |  |
|  | *qnrS* | qnrS-F | GCA AGT TCA TTG AAC AGG GT | Cattoir *et al. 2007* | PCR screening |
|  |  | qnrS-R | TCT AAA CCG TCG AGT TCG GCG |  |  |
|  | *aac(6')-Ib* | aac(6´)Ib-F | TTG CGA TGC TCT ATG AGT GGC TA | Park *et al.* 2006 | PCR screening/PCR for Sanger sequencing |
|  |  | aac(6´)Ib-R | CTC GAA TGC CTG GCG TGT TT |  |  |
|  | *qepA* | qepA-F | TGG TCT ACG CCA TGG ACC TCA | Karczmarczyk *et al.* 2010 | PCR screening/PCR for Sanger sequencing |
|  |  | qepA-R | TGA ATT CGG ACA CCG TCT CCG |  |  |
|  | *oqxA* | oqxAF | CTC GGC GCG ATG ATG CT | Kim *et al.* 2009 | PCR screening/PCR for Sanger sequencing |
|  |  | oqxAR | CCA CTC TTC ACG GGA GAC GA |  |  |
|  | *oqxB* | oqxBs | TTC TCC CCC GGC GGG AAG TAC |  | PCR screening/PCR for Sanger sequencing |
|  |  | oqxBa2 | CTC GGC CAT TTT GGC GCG TA |  |  |
| **Tetracyclines** | *tet*(A) | tetA-F | GCT ACA TCC TGC TTG CCT TC | Ng *et al.* 1999 | PCR screening |
|  |  | tetA-R | CAT AGA TCG CCG TGA AGA GG |  |  |
|  | *tet*(B) | tetB-F | TTG GTT AGG GGC AAG TTT TG | Ng *et al*. 1999 | PCR screening |
|  |  | tetB-R | GTA ATG GGC CAA TAA CAC CG |  |  |
|  | *tet*(D) | tetD-F | AAA CCA TTA CGG CAT TCT GC | Ng *et al*. 1999 | PCR screening |
|  |  | tetD-R | GAC CGG ATA CAC CAT CCA TC |  |  |
| **Amphenicols** | *catA1* | cat-F | CCT GCC ACT CAT CGC AGT | Faldynova *et al.* 2003 | PCR screening |
|  |  | cat-R | CCA CCG TTG ATA TAT CCC |  |  |
|  | *cmlA* | cmlA-F | TGT CAT TTA CGG CAT ACT CG | Saenz *et al.* 2004 | PCR screening |
|  |  | cmlA-R | ATC AGG CAT CCC ATT CCC AT |  |  |
|  | *floR* | floR-F | GCG ATA TTC ATT ACT TTG GC | Faldynova *et al.* 2003 | PCR screening |
|  |  | floR-R | TAG GAT GAA GGT GAG GAA TG |  |  |
| **Aminoglycosides** | *strA* | strA/F | CCT ATC GGT TGA TCA ATG TC | Faldynova *et al*. 2003 AAC 47, 2002-2005 | PCR screening |
|  |  | strA/R | GAA GAG TTT TAG GGT CCA CC |  |  |
|  | *aac(3)-II* | aac3-II-F | CAA TAA CGG AGG CAA TTC G | Soge *et al.* 2006 | PCR screening |
|  |  | aac3-II-R | GAT TAT CAT TGT CGA CGG |  |  |
|  | *aac(3)-IId* | aac3II-d_F | CTG TGA TGG GAT ACG CGT CG | Leflon-Guibout *et al.*  2004 | PCR screening |
|  |  | aac3-II-R | GAT TAT CAT TGT CGA CGG |  |  |
| **Sulphonamides** | *sul1* | sul1-F | CTT CGA TGA GAG CCG GCG GC | Zhao *et al.* 2001 | PCR screening |
|  |  | sul1-R | GCA AGG CGG AAA CCC GCG CC |  |  |
|  | *sul2* | sul2-F | AGG GGG CAG ATG TGA TCG AC | Faldynova *et al.* 2003 | PCR screening |
|  |  | sul2-R | GCA GAT GAT TTC GCC AAT TG |  |  |
|  | *sul3* | sul3-F | GAG CAA GAT TTT TGG AAT CG | Perreten and Boerlin 2003 | PCR screening |
|  |  | sul3-R | CAT CTG CAG CTA ACC TAG GGC TTT GGA |  |  |
| **Integrons** | *intI1* | int1-F | CCT CCC GCA CGA TGA TC | Zhao *et al.* 2001 | PCR screening for genes encoding integrase of class 1 integrons |
|  |  | int1-R | TCC ACG CAT CGT CAG GC |  |  |
|  | *intI2* | int2-F | CAC GGA TAT GCG ACA AAA AGG T | Saenz *et al.* 2004 | PCR screening for genes encoding integrase of class 2 integrons |
|  |  | int2-R | GTA GCA AAC GAG TGA CGA AAT G |  |  |
|  | class 1 integron | 5´CS | GGC ATC CAA GCA GCA AG | Faldynova *et al.* 2003 | PCR for RFLP and/or Sanger sequencing |
|  |  | 3´CS | AAG CAG ACT TGA CCT GA |  |  |
|  | class 2 integron | Hep74 | CGG GAT CCC GGA CGG CAT GCA CGA TTT GTA | White *et al.* 2001 | PCR for RFLP and/or Sanger sequencing |
|  |  | Hep51 | GAT GCC ATC GCA AGT ACG AG |  |  |
|  |  | cib/R | CAG AAT ATT GCG TTT ATA GTC C |  |  |

**Supplemental Table 1 References**

Agersø Y, Aarestrup FM, Pedersen K *et al*. Prevalence of extended-spectrum cephalosporinase (ESC)-producing *Escherichia coli* in Danish slaughter pigs and

retail meat identified by selective enrichment and association with cephalosporin usage. *J Antimicrob Chemother* 2012; **67:**582-8.

Brinas L, Zarazaga M, Saenz Y *et al.* Beta-lactamases in ampicillin-resistant *Escherichia coli* isolates from foods, humans, and healthy animals. *Antimicrob*

*Agents Chemother* 2002; **46**:3156-63.

Cavaco LM, Hasman H, Xia S *et al*. *qnrD*, a novel gene conferring transferable quinolone resistance in *Salmonella enterica* serovar Kentucky and

Bovismorbificans strains of human origin. *Antimicrob Agents Chemother* 2009; **53**:603-8.

Cattoir V, Poirel L, Rotimi V *et al.* Multiplex PCR for detection of plasmid-mediated quinolone resistance *qnr* genes in ESBL-producing enterobacterial isolates.

*J Antimicrob Chemother* 2007; **60**:394-7.

Park CH, Robicsek A, Jacoby GA et al. Prevalence in the United States of *aac(6 ')-Ib-cr* encoding a ciprofloxacin-modifying enzyme. *Antimicrobial Agents*

*and Chemotherapy* 2006; **50**: 3953-5.

Faldynova M, Pravcova M, Sisak F *et al.* Evolution of antibiotic resistance in *Salmonella* enterica serovar typhimurium strains isolated in the Czech Republic

between 1984 and 2002. *Antimicrob Agents Chemother* 2003; **47**:2002-5.

Karczmarczyk M, Martins M, McCusker M *et al.* Characterization of antimicrobial resistance in *Salmonella enterica* food and animal isolates from Colombia:

identification of a *qnrB19*-mediated quinolone resistance marker in two novel serovars. *FEMS Microbiol Lett* 2010; **313**:10-9.

Kim BH, Park CH, Kim CJ *et al.* Prevalence of plasmid-mediated quinolone resistance determinants over a 9-year period. *Antimicrob Agents Chemother* 2009;

**53**:639-45.

Kim BH, Wang M, Park CH *et al. oqxAB* encoding a multidrug efflux pump in human clinical isolates of Enterobacteriaceae. *Antimicrob Agents Chemother*

2009; **53**:3582-4.

Leflon-Guibout V, Jurand C, Bonacorsi S *et al.* Emergence and spread of three clonally related virulent isolates of CTX-M-15-producing *Escherichia coli*

with variable resistance to aminoglycosides and tetracycline in a French geriatric hospital. *Antimicrob Agents Chemother* 2004; **48:**3736-42.

Lewis JS, II, Herrera M, Wickes B *et al.* First report of the emergence of CTX-M-Type extended-spectrum beta-lactamases (ES-BLs) as the predominant ESBL

isolated in a US health care system. *Antimicrob Agents Chemother* 2007; **51**:4015-21.

McGettigan SE, Hu B, Andreacchio K et al. Prevalence of CTX-M beta-lactamases in Philadelphia, Pennsylvania. *J Clin Microbiol* 2009; **47:**2970-4.

Ng LK, Mulvey MR, Martin I *et al.* Genetic characterization of antimicrobial resistance in Canadian isolates of *Salmonella* serovar typhimurium DT104.

*Antimicrob Agents Chemother* 1999; **43**:3018-21.

Perez-Perez FJ, Hanson ND. Detection of plasmid-mediated AmpC beta-lactamase genes in clinical isolates by using multiplex PCR. *J Clin Microbiol* 2002;

**40**:2153-62.

Perreten V, Boerlin P. A new sulfonamide resistance gene (*sul3*) in *Escherichia coli* is widespread in the pig population of Switzerland. *Antimicrob Agents*

*Chemother* 2003; **47**:1169-72.

Pitout JD, Hossain A, Hanson ND. Phenotypic and molecular detection of CTX-M-beta-lactamases produced by *Escherichia coli* and *Klebsiella* spp. *J Clin*

*Microbiol* 2004; **42**:5715-21.

Robicsek A, Strahilevitz J, Sahm DF *et al.* *qnr* prevalence in ceftazidime-resistant Enterobacteriaceae isolates from the United States. *Antimicrob Agents*

*Chemother* 2006; **50**:2872-4.

Saenz Y, Brinas L, Dominguez E *et al.* Mechanisms of resistance in multiple-antibiotic-resistant *Escherichia coli* strains of human, animal, and food origins.

*Antimicrob Agents Chemother* 2004; **48**:3996-4001.

Soge OO, Queenan AM, Ojo KK *et al.* CTX-M-15 extended-spectrum β-lactamase from Nigerian *Klebsiella pneumoniae*. *J Antimicrob Chemother* 2006; **57:**24-30.

Sturenburg E, Kuhn A, Mack D *et al.* A novel extended-spectrum beta-lactamase CTX-M-23 with a P167T substitution in the active-site omega loop associated

with ceftazidime resistance. *J Antimicrob Chemother* 2004; **54**:406-9.

White PA, McIver CJ, Rawlinson WD. Integrons and gene cassettes in the Enterobacteriaceae. *Antimicrob Agents Chemother* 2001; **45**:2658-61.

Zhao SH, White DG, Ge BL *et al.* Identification and characterization of integron-mediated antibiotic resistance among shiga toxin-producing *Escherichia coli*

isolates. *Appl Environ Microbiol* 2001; **67**:1558-64.
